# MANTRA: development and localization of a mobile educational health game targeting low literacy players in low and middle income countries

**DOI:** 10.1186/s12889-020-09246-8

**Published:** 2020-07-28

**Authors:** Sonja Mueller, Delphine Soriano, Andrei Boscor, Naomi Saville, Abriti Arjyal, Sushil Baral, Maureen Fordham, Gareth Hearn, Virginie Le Masson, Rachya Kayastha, Patty Kostkova

**Affiliations:** 1 Institute for Risk and Disaster Reduction, University College London, Gower Street, London, WC1E 6BT UK; 2 Centre for Digital Public Health in Emergencies (dPHE), University College London, Gower Street, London, WC1E 6BT UK; 3 Institute for Global Health, University College London, 30 Guilford Street, London, WC1N 1EH UK; 4 Health Research and Social Development Forum, Prasuti Griha Marg, Kathmandu, 44600 Nepal; 5 Centre for Gender and Disaster, University College London, Gower Street, London, WC1E 6BT UK; 6 Hearn GeoServe, Ltd, London, UK; 7 Overseas Development Institute, 203 Blackfriars Road, London, SE1 8NJ UK

**Keywords:** Serious game, Educational game, mHealth

## Abstract

**Background:**

Mobile technology is increasingly important for delivering public health interventions to remote populations. This research study developed, piloted, and assessed a serious game for mobile devices that teaches geohazard, maternal, and neonatal health messages. This unique mHealth intervention aimed at low-literacy audiences in low resource settings is part of the Maternal and Neonatal Technologies in Rural Areas (MANTRA) project: Increasing maternal and child health resilience before, during, and after disasters using mobile technology in Nepal.

**Methods:**

The serious game was developed through a co-creation process between London and Kathmandu based researchers by email and video-calling, and face-to-face with local stakeholders in Nepal. The process identified core needs, developed appropriate pictograms and mechanics, and tailored the pilot serious game to the local cultural context. Evaluations and feedback from end users took place in rural villages and suburban Kathmandu in Province Three. Field evaluation sessions used mixed methods. Researchers observed game play and held focus group discussions to elicit qualitative feedback and understand engagement, motivation, and usability, and conducted a paired pre- and post-game knowledge assessment.

**Results:**

The MANTRA serious game is contextualized to rural Nepal. The game teaches 28 learning objectives in three modules: maternal health, neonatal health, and geohazards, through picture matching with immediate audio and visual feedback. User feedback from focus groups demonstrated high engagement, motivation, and usability of the game.

**Conclusions:**

This MANTRA study is a unique mHealth intervention of a serious game to teach core health and geohazards messages to low-literacy audiences in rural Nepal. Although the mobile game is tailored for this specific context, the developmental process and insights could be transferable to the development of other games-based interventions and contextualized for any part of the world. Successfully targeting this low-literacy and illiterate audience makes the MANTRA development process the first of its kind and a novel research endeavor with potential for widespread impact and adoption following further game development.

**Trial registration:**

This project was approved by the University College London Ethics Committee in London, United Kingdom [10547/001], and the Nepal Health Research Council in Kathmandu, Nepal [Reg. No. 105/2017]. All participants provided informed written consent.

## Background

The 2015 Nepal earthquake isolated communities and reduced access to medical advice and healthcare services, including vital support for vulnerable populations like pregnant and perinatal women and their newborns [1, 2]. mHealth, defined as the use of mobile and wireless technologies for health [3], can help overcome obstacles to public health initiatives in times of disaster and crisis as well as day-to-day exposure to hazards, remote populations, rough terrain, and limited distribution channels [4, 5].

The project in Nepal entitled “Maternal and Neonatal Technologies in Rural Areas (MANTRA): Increasing maternal and child health resilience before during and after disasters using mobile technology” investigated building women’s resilience by improving access to information and communications before, during, and after environmental disasters by developing an mHealth intervention to support and expand existing participatory learning public health interventions, social protection mechanisms, and awareness of everyday geohazards [6–12].

mHealth is rapidly expanding in health education, including low and middle income countries (LMICs) [13]. These interventions are economically attractive options due to scalability and ease of modifying or adding content compared to traditional and face to face methods for distributing health campaigns and messages to the public in low income settings [14, 15]. Serious games, aimed at conveying educational knowledge rather than mere entertainment [16], are a type of intervention within mHealth.

The authors did not find any publication on a serious game targeted to a low-literacy adult audience in LMICs and covering maternal health, neonatal health, and geohazards topics. Two of the largest serious game interventions aimed at low-literacy audiences, although in a high income setting, are the eBug and edugames4all projects, which are for children [17–19]. Lessons for developing serious games according to the needs of low-literacy users could be transferred from research aimed at other illiterate audiences, such as toddlers and young children. One example is a game consisting solely of images, animation, and audio, called “Listening with Lemur”, which is for children aged one and a half to 3 years with recent cochlear implants [20]. Such a game is text-free and image based, with simple game mechanics and structure, and designed for a specific user group. This makes the game ideal for young children and illiterate users unfamiliar with smartphones. These related projects highlight the importance of co-creation, iterative development, and focus groups with the target audience, to gain qualitative feedback to ensure that games match audience capabilities.

More research and evidence is needed to fully explore the challenges of implementing a serious game for health in a low resource setting. Kostkova succinctly presents the challenges facing the digital health sector and the huge potential to impact the health sector, especially in LMICs [13]. Serious games for health are no exception to these challenges or potential, and research into the needs, challenges, and existing health systems in a region or nation is required to ensure the technology is useful and accepted by patients, communities, health workers, and healthcare systems [13, 21].

The MANTRA project study area is rural Nepal. Nepal is a suitable study area for piloting an mHealth intervention because of rising access to mobile phones [22, 23], risks inherent to a dispersed population in a geologically active region [6, 24], and vulnerabilities at national, community and individual scales [25]. Communication links have been strengthened as the number of mobile phone subscriptions in Nepal reached 110 subscriptions per 100 people in 2016 [23], growing from about 9 million subscriptions in 2010 to over 32 million in 2016 [22]. Nepal’s Demographic and Health Survey 2016 reports that mobile phone ownership is highest among the 20–24 age group for both women and men, at 85 and 96% respectively [25]. Rising access to mobile phones supports the use of mobile health tools to reach a population.

In Nepal, primary healthcare is largely delivered by rural health workers at local health facilities, with outreach conducted by Female Community Health Volunteers (FCHVs) to provide healthcare education their local area (often c.1000 population) [26]. In a disaster situation like the 25 April, 2015 earthquake, broken transportation links isolated communities from healthcare and medical advice [2, 27]. Kavrepalanchok district was one of 14 districts highly affected by the 2015 and accessible for research, so study locations were selected from this district, now within Province 3 [2, 27].

The Nepal context shares many similarities and barriers to other LMICs, like a large rural population, a large illiterate adult population, and rising mobile phone ownership [22, 24, 28]. Many of the lessons learned through the MANTRA development process could be transferable to other LMIC settings.

From this research context, we developed a serious game. Our objective in presenting the development process for the MANTRA serious game is to offer insight and a pathway for future research and implementation of similar mHealth interventions targeting a low-literacy audience. We incorporate best practice for serious games in low-resource settings by working towards low cost, geographical scalability, easy localization to new countries and cultural settings, and engagement to deliver important public health messages [3, 4, 13, 29–31]. By contributing to the growing body of research for mHealth interventions, this research aims to share a novel, transferable process to build a game for low-literacy populations. The value of presenting this research is in sharing a transferable process for developing mHealth interventions and localizing an exciting mHealth product specifically to a low literacy audience, challenging the usual settings and methods for engaging this hard-to-reach target audience.

## Methods

To present this novel serious game aimed at low-literacy audiences about maternal health, neonatal health, and geohazards, the remainder of the article is structured into four parts. Methods details the process of designing and contextualizing the MANTRA serious game. Results presents the MANTRA serious game. Discussion reflects on lessons learned by the research team and the successes, practicalities, and opportunities to improve the process for subsequent iterations of the MANTRA game and other studies. The Conclusions section suggests value of this process and the transferability of insights and the localization and contextualization process.

**Fig. 1:**
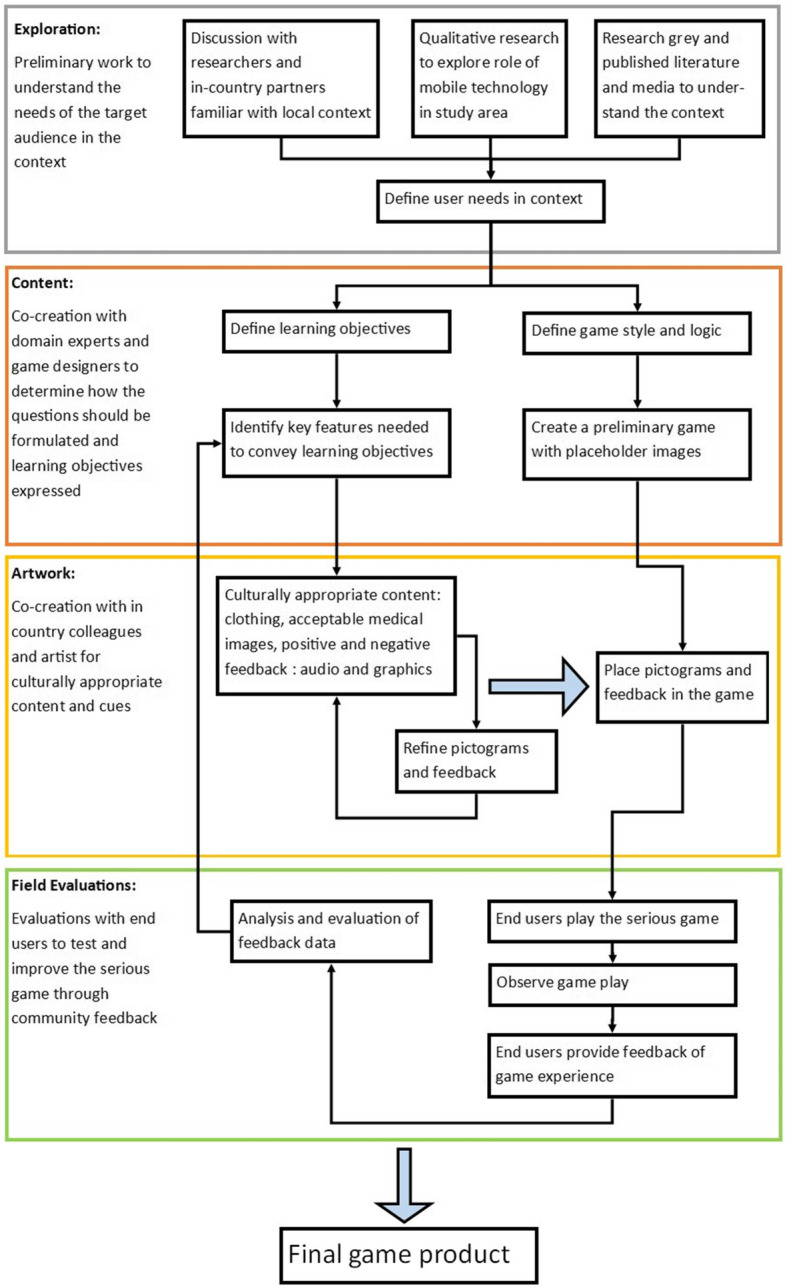
Process Workflow diagram. The four colored boxes divide the methodology into four major process stages, Exploration is outlined in grey, Content in orange, Artwork in yellow, and Field Evaluations in green. The black arrows indicate progression through the methodology, and the larger blue arrows indicate exiting an iterative design cycle. Cycles within the methodology are 1) co-creating the artwork and 2) addressing evaluations and feedback from the field evaluation sessions

Co-creation and co-design were guiding principles throughout the design process. Co-creation is “collective creativity” and co-design specifies that this principle “is applied across the whole span of a design process” [32], bringing decision-making, power, and stake in the end product [33]. Additional considerations were setting priorities based on user needs, input from domain experts, iterative co-design for localization and contextualization, and controlled environment testing [30, 34, 35]. The game design methodology builds on these guiding principles and typical software development processes [34, 36, 37]. Co-creation discussions throughout the project were conducted through video calling and collaborative online workspaces. The method is divided into four stages, illustrated as four colored boxes in Fig. 1.

### Process workflow

#### Explore

The grey ‘Exploration’ box begins the methodology with research into the needs and context of the users and wider setting. Other components of the MANTRA research program were helpful to understand the target audience experience related to maternal and neonatal health, the 2015 earthquake, and mobile technologies.

Initial research for the game, context, and user needs included previous work in Nepal by coauthors and Health Research and Social Development Forum (HERD) and research on mobile phone use and telecommunication infrastructure. Most importantly, a field trip in March 2017 organized by HERD assessed local conditions, studied site needs, and preliminary technical constraints to inform the serious game design, context, and artwork. To supplement these game-focused explorations, a few questions on mobile phone interest and use were included in focus group discussions for 5 groups and about 60 qualitative interviews to provide context from the target audience and potential end users of the serious game. However, these qualitative studies were primarily about experience of maternal and perinatal health in earthquake disasters with health workers, women, and support people (in preparation). Discussions among the research team built an understanding of end user needs and cultural context, based in research expertise and experience living in Nepal. This foundation informed the serious game design and artwork.

#### Content

The orange ‘Content’ box builds on the exploration step with domain experts defining learning objectives and working with game designers to choose game mechanics suited to presenting learning objectives to users. Learning objectives are specific pieces of information or concepts within the topic, or module, that support broad project goals [18, 38–40]. Game mechanics are the rules, procedures, and essential interactions that create a meaningful game [38, 41], and game structure encompasses the construction of questions, feedback, and progression mechanisms in the game.

Game mechanics were informed by the exploration stage insight that many end users in the target audience were unlikely to be familiar with smartphones. User needs of the target audience drove the selection and design of the game mechanics, so the MANTRA serious game has a simple structure and intuitive game mechanics. Ultimately, we selected a ‘drag and drop’ multiple-choice format with immediate feedback and accumulating leaves and flowers as progress mechanisms from a range of options including binary questions (yes/no), story-based, and facts by rote learning, etc. We developed both a touchscreen tutorial and a training level to accompany the game, since a training level for complex games (such as storytelling mechanics or problem based learning) can help players with little familiarity of digital games or lower reading skills [36, 42, 43]. The game has three levels of increasing complexity to allow players to repeatedly see and interpret the learning objectives, since learning is reinforced by repeatedly seeing the content and receiving immediate feedback on correct or incorrect answers [18, 44].

The learning objectives are represented by small pictograms to be accessible to the low-literacy target audience. The MANTRA learning objectives were co-designed by HERD and domain experts, incorporating research from the exploration stage [7, 45–47]. These learning objectives were built upon picture cards developed by Mother and Infant Research Activities (MIRA) and University College London for previous participatory learning and action women’s group trials on neonatal mortality in Nepal [11, 48, 49]. They also drew upon neonatal sepsis management guidelines for FCHVs as tested in the Nepal plains [11, 50, 51]. Then, the co-authors (NS, GH) and artist (DS) distilled key features of each learning objective for pictogram development.

#### Artwork

The yellow ‘Artwork’ box highlights the first cyclic iterative process of the artist, domain experts, and in country partners creating abstract images, or pictograms, to convey learning objectives to the target audience, with informal input from in country colleagues on the suitability of artwork styles, logic, and symbols. These initial pictograms were placed in the game and tested within the research team to build an initial version of the game to evaluate with end users. In country partner HERD was invaluable in localization and contextualization of the game and artwork to the specific culture and setting of Kavrepalanchok district, now part of Province Three, in Nepal. Artwork was redesigned after the field evaluations to incorporate feedback and suggestions from the user evaluations.

Nepal-based co-authors advised suitable graphics and sounds to convey good and positive feedback or bad and negative feedback, clothing for women and infants, clues for scale in geohazard pictograms, and to convey danger. The co-creation process for localization and contextualization was realized through frequent dialogue via emails and video calling between the UK based researchers building the artwork and game mechanics and the Nepal based researchers advising from their personal and professional experience living in Nepal.

#### Field evaluations

The next step is field testing with end users in the target audience. The green ‘Field Evaluations’ box contains steps with end users to evaluate the game for design, user experience, and knowledge gain through observations and feedback. Then, the second cycle illustrated in Fig. 1 continues, returning to the content and artwork cycle to include community end user input in the next version of the game.

We completed two iterations of this cycle, with two mixed methods field evaluations with end users in ten sessions. Sessions consisted of game play observations, focus group discussions about the game experience, and a paired pre- and post- game knowledge assessment. Subjects were recruited using the chain-referral method [52]. HERD researchers asked contacts in the villages to request FCHVs and other community members to participate in the study. Most sessions consisted of female participants although one session consisted of men in the community to test their attitude and acceptance of the game. The first evaluation cycle in October 2017 consisted of 6 sessions, and the second cycle in November 2017 had 4 sessions, averaging 7.1 participants in a session. Participant characteristics are briefly presented in section 3.3.1, discussed in greater detail in Mueller et al. (in preparation) [53], and focus group methodology in Kayastha [54]. In each session, researchers used two methods to evaluate the user experience. The first was observation during game sessions supported by video recordings of participants playing the game. The second method was focus group discussions to bring the target audience into the co-creation process, gain feedback on the game, and understand the user experience, such as likes and dislikes about the game and artwork, clarify confusing messages, and gather suggestions for future versions of the serious game (topic guide in Table 1). Focus group discussions were facilitated by HERD researchers, audio recorded in Nepali, transcribed and translated to English for the game designers to read and incorporate end user feedback into the game design. A paired pre- and post-game knowledge assessment was also part of these sessions to measure knowledge gain from playing the game by McNemar analyses and paired T-tests (52, in preparation).

**Table 1:** Topic guide for evaluation focus group discussions

1. Did you experience the symptoms or signs in the game?	
2. Did you experience any symptoms or signs that were not in the game?	
3. What new things (if any) did you learn from playing the game?	
a. About maternal and newborn health	
b. About natural hazards	
4. What do you think of the mobile phone game?	
a. What did you like about the game?	
b. What did you not like?	
c. What things did you find easy to understand?	
i. About maternal and newborn health	
ii. About natural hazards	
d. What did you find difficult to understand or confusing?	
5. If this game were available on a mobile phone for you to play, how do you think you would use the mobile phone game?	
6. Did you feel motivated to keep playing? Why?	
7. How do you feel it would change your behaviour	
a. With respect to maternal and newborn health	
b. With respect to natural hazards	
8. How do you think that playing the mobile phone game would affect your decision making when faced with a problem	
a. Concerning maternal or newborn health	
b. Concerning a natural hazard	
9. Do you feel empowered by playing this game?	
10. What would you tell others about this game?	
11. Would it be more suitable for a particular group? For example?	
a. Probe about age group / wealth group / educational statue / gender group / occupation	
12. How else could this mobile game be applied in your community?	
13. What health issues worried you about	
a. pregnancy and childbirth?	
b. Your newborn baby?	
14. When would you seek medical help?	
a. During pregnancy	
b. For your newborn baby??	
c. If no	
i. What health issues worry you about a future:	
1. pregnancy and childbirth?	
2. newborn baby?	
ii. When would you seek medical help?	
1. During pregnancy?	
2. For your newborn baby?	
15. Where do you get health information?	
16. If FCHV: Do you communicate with other FCHVs through phones? How?	
17. Do you use your phones in emergencies?	
a. How?	
b. Medical emergency?	
c. Natural hazard event?	

### Iterations

We successfully completed two iterations of field evaluations, essential steps to evaluate the pilot version of the serious game. Our longer-term plan is for iterations of content, artwork, and field evaluations to continue and test future versions of the game product with a wider user base, and eventually liaise with appropriate authorities for approval and distribution. This future work is detailed in the Discussion section.

### Ethics approval and consent to participate

The MANTRA project research studies were approved by the University College London Ethics Committee in London, United Kingdom [10,547/001] and the Nepal Health Research Council in Kathmandu, Nepal [Reg. No. 105/2017]. HERD co-authors and researchers explained the research to participants and all focus group and interview participants provided informed written consent. The funding body played no role beyond the funding call in the design of the study, data collection, analysis, data interpretation, or writing the manuscript.

### Technical aspects

Each digital intervention has to take into account technical constraints in the setting. We considered available telecommunications publications and data, in country colleagues and partners, and available devices, local mobile phone infrastructure, availability and reliability of data networks as well as availability of electricity infrastructure for charging devices.

The MANTRA game intervention was built in Unity game framework [55] for Android, iOS, and Windows mobile devices. For field testing, the game was installed on six Samsung Galaxy 7 smartphones to minimize the chance of variation and unforeseen problems. Since mobile data networks are limited in rural areas of Nepal and users may not be able to afford to pay for data to play online, an internet connection is not required to play the game [25, 56]. Later, when internet was available, game data was uploaded to a MongoDB back end database running on a Heroku server [57, 58].

## Results

### Game mechanics

**Fig. 2:**
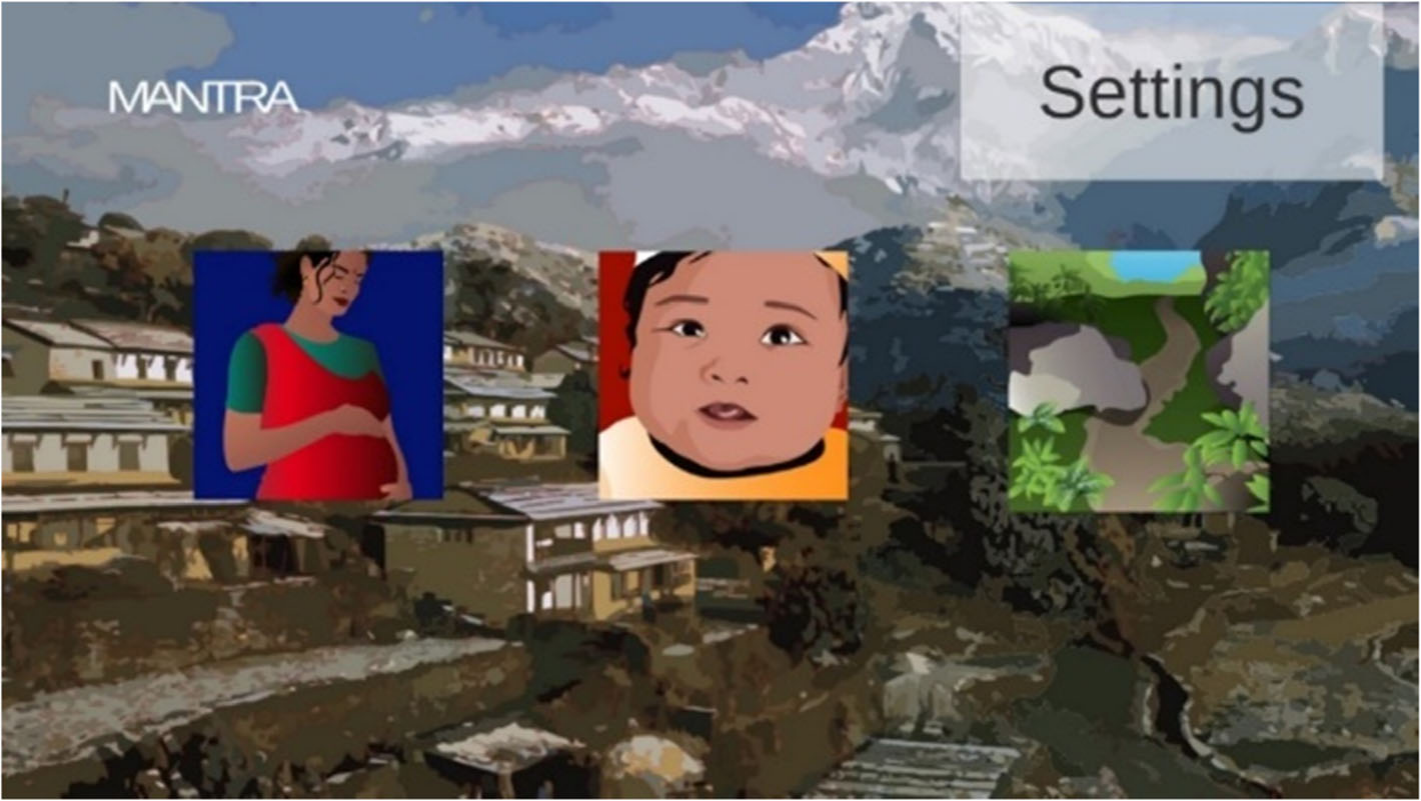
Three modules displayed on the home screen of the MANTRA game intervention. From left to right, the modules are maternal health, neonatal health, and geohazards

Modules were chosen based on the project goals and to assemble learning objectives into thematic groups for focused teaching of maternal health, neonatal health, and geohazards. In the game intervention, players select which module they wish to complete from the home screen as shown in Fig. 2.

#### Touchscreen tutorial

**Fig. 3:**
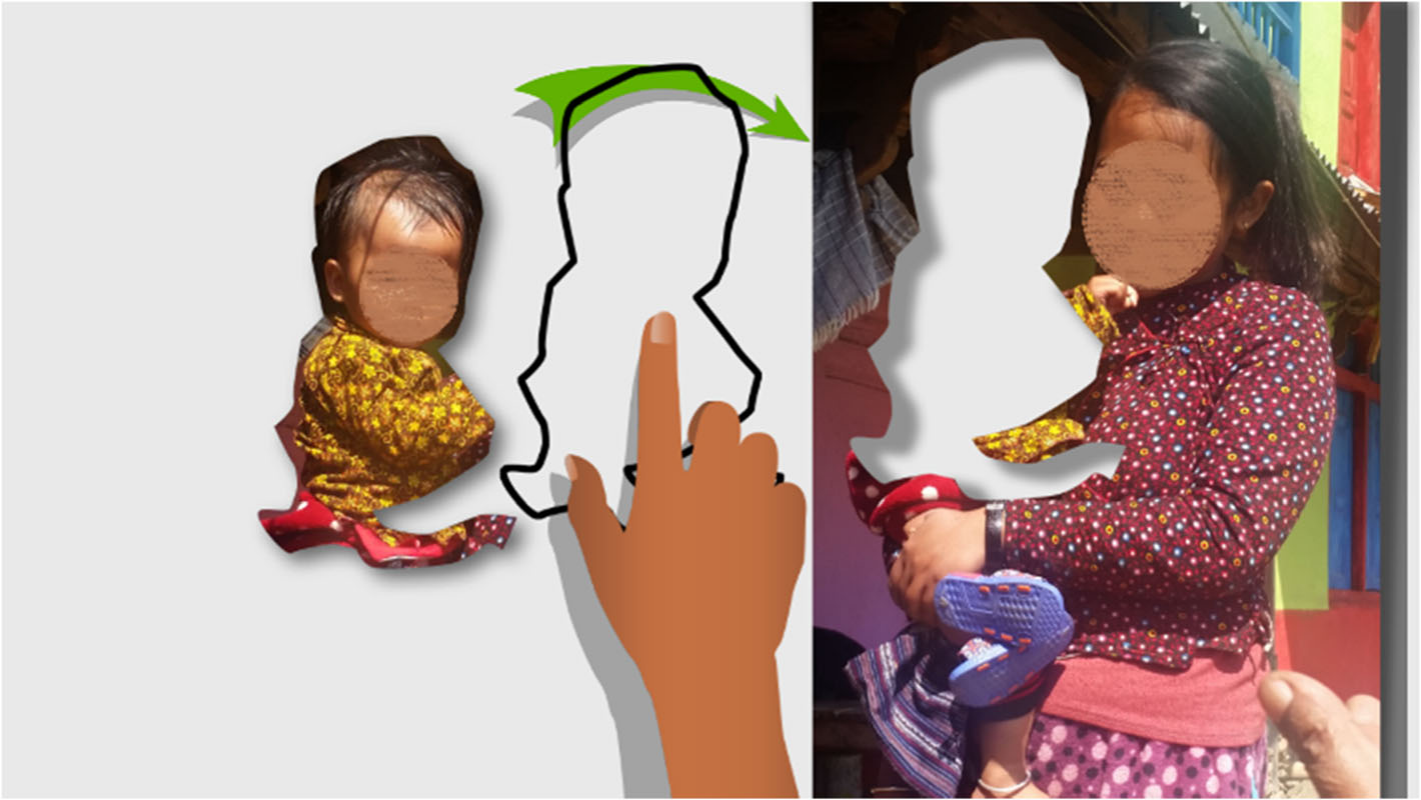
Screen of the tutorial on drag and drop on a touch screen interface. This screenshot of the animation shows the animated hand giving a hint to the user of how to place their hand to move the baby across the screen. The photograph was taken at the first exploratory visit to the village and added to help participants identify with the content of the game. Consent for use of images was obtained and is held by HERD

A touch screen tutorial was developed and included in the start of user sessions to help players unfamiliar with smartphones to understand a touch screen interface. For simplicity, the‘drag and drop’ mechanism is the only action needed in the game. Figure 3 is a screenshot of the hint that appears in the MANTRA tutorial if the user has not moved the baby cutout into the photograph after a short time. An animated hand moves the baby outline across the screen into the gap in the photograph. Then, the player has another opportunity to try the ‘drag and drop’ interaction.

#### Training level

**Fig. 4:**
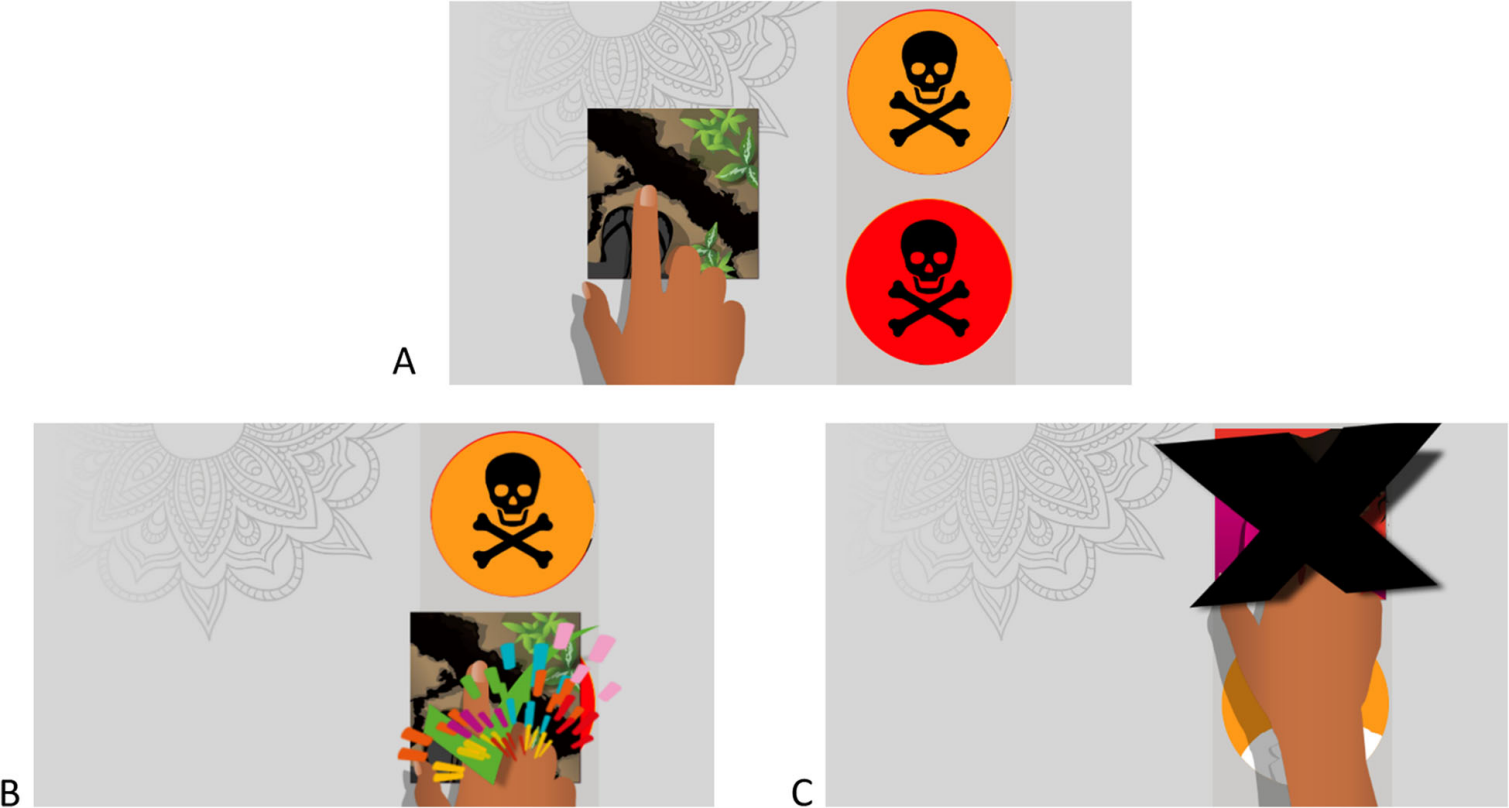
Screenshots illustrate sample questions from the tutorial on how to ‘answer’ questions to play the game by dragging tiles. **a** shows the initial layout of the question. **b** illustrates the positive feedback graphic when a question is answered correctly, which would also be accompanied by birdsong. **c** illustrates the negative feedback graphic of a large black X and is accompanied by thunder

Following the touchscreen tutorial, a training video of animated example questions demonstrated expected user interactions, the concept that each learning objective tile (square) is associated with a response tile (circle), and to drag their chosen response tile across the screen. Like in the tutorial, an animated hand drags a learning objective tile across the screen to a response tile as pictured in Fig. 4. Initially, the training level of the MANTRA intervention was outside the game. In the November evaluation sessions, the training level was integrated into the game intervention to automatically appear after the touchscreen tutorial in a new user session to make the flow easier for a new user.

#### Increasing complexity, repetition, and error pool

**Fig. 5:**
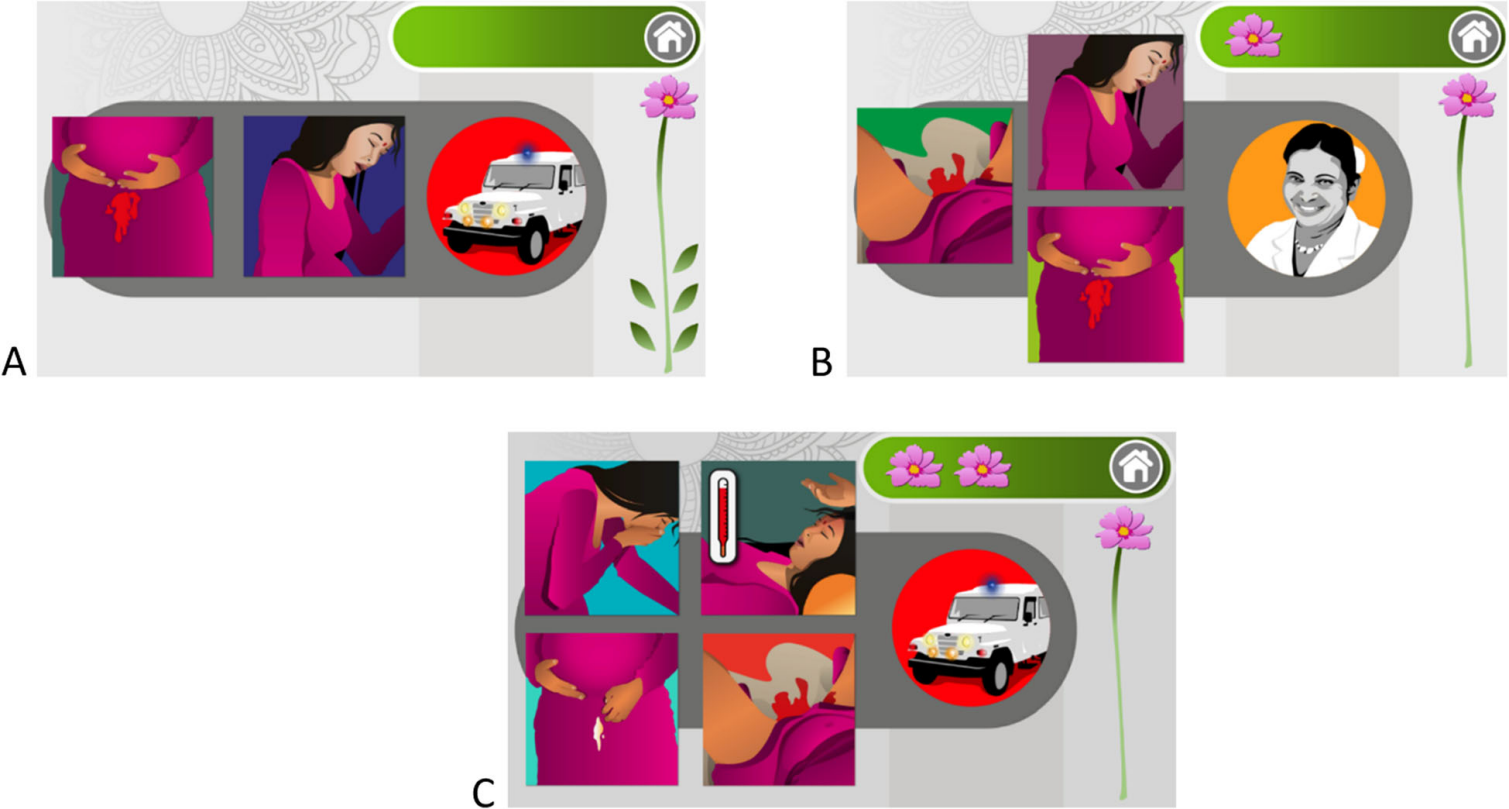
Screens from the MANTRA serious game. **a** A question in Level 1 asking which situation requires emergency care. **b** A question from Level 2 with three choices for a less urgent condition. **c** A question from level 3 asks which of the four choices requires emergency care

Each module contains three levels of complexity (Fig. 5), allowing players to repeatedly see and interpret the learning objectives. Questions are formed by selecting a learning objective with its paired urgency pictogram, which will be the correct answer, then randomly selecting one to three other learning objectives of the opposite urgency or risk for the incorrect answer(s) depending on the level. Once a question was successfully answered, the learning objective was not selected to be a correct answer again in that level (but is shown again in higher levels). If a question was answered incorrectly, the correct learning objective was added to an error pool and shown up to three times throughout the level. The player continues the level until all learning objectives are seen and either a single learning objective is answered incorrectly three times and the player ‘fails’ the level, or the error pool contains only two learning objectives and the player ‘passes’ the level, progressing to the next level.

#### Game feedback and progression

Immediate feedback to each question is given to reinforce learning, a standard method in games to improve learning outcomes. If the user correctly matches the learning objective and response, the feedback is a green checkmark and fireworks accompanied by an audio clip of birdsong, while an incorrect match results in a black X accompanied by an audio clip of thunder.

Two scores show progression through the game. Within a level, each correct answer adds a leaf to a flower stem as a score as seen on the right side of the screenshots in Fig. 5. When the player has accumulated enough leaves, the level is successfully completed and a large flower appears with fireworks and birdsong. These flowers accumulate during a single user session, and appear at the top right of the screen as in Fig. 5.

### Learning objectives and artwork

**Fig. 6:**
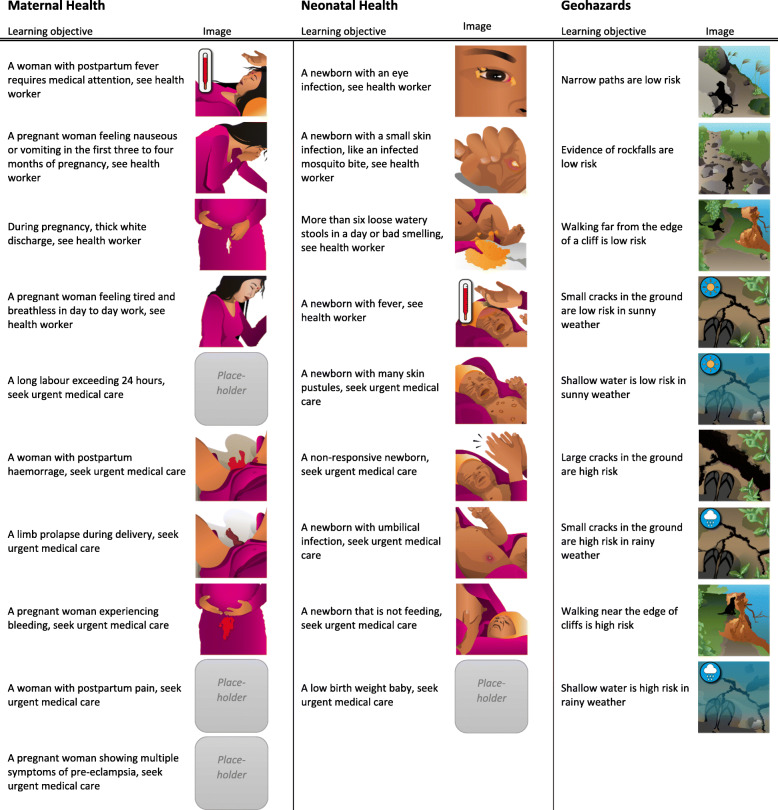
Learning objectives categorized by module. The image accompanying each learning objective is the designed image or placeholder image as designed for the November field evaluation sessions. Placeholder images were adapted from existing Mother and Infant Research Activities (MIRA) intervention materials [11, 48–51] and will be replaced in subsequent versions of the game [59]

To reach a low-literacy population, the learning objectives were presented as small pictograms illustrating landscape scenes or medical conditions that were suitable for display on a smartphone screen. The co-designed pictograms used in the November field evaluation sessions are illustrated in Fig. 6.

#### Artwork

Tailoring the artwork to the local culture was successful since focus group participants said the pictured women and infants reflect themselves and their community, the colors are beautiful, and they like the positive progress and feedback notifications.

#### Localization, contextualization and cultural appropriateness

The MANTRA serious game has numerous examples of localization and contextualization. For instance, women in the artwork were depicted wearing pink, since married Nepali women wear pink and red, and infants wear a hat for warmth. Roaming dogs are a common sight in Nepal, so they were incorporated as a clue for scale in geohazard pictograms. The birdsong and thunder audio clips and progress flowers were selected since the study areas were primarily rural villages and our HERD colleagues recommended that these would be understood as ‘positive’ and ‘negative’ in Nepali culture. In focus group discussions, participants mentioned a red, yellow, and green system to note progressive danger that they observed in a tape measuring tool to measure childrens’ arms to indicate malnutrition, which we incorporated into the game color schemes to represent danger and risk.

### Evaluation and the user experience

#### Participant characteristics

Thirty-six of 71 participants reported owning smartphones. The 71 participants across four locations had education ranging from informal to a bachelor’s degree and an average age of 40 years.

#### Observations of game play

During the MANTRA pilot study, researchers observed many players talking and joking with each other during game sessions despite facilitators asking participants to avoid discussion while playing the game and to do their best without assistance. People preferred to interact with others while playing. Another insight into the game logic was that a question could be answered twice, as several players discovered they could quickly replay a question during the immediate negative feedback to answer correctly and receive positive feedback of fireworks and birdsong. Observations also indicated that the ‘drag and drop’ touch screen tutorial may need expansion, since several participants struggled with the interaction until they were assisted by a facilitator or fellow participant.

#### Focus group discussions

Study participants suggested supplementing tutorial and teaching levels with audio to provide verbal instructions about game play and to communicate information that may not be easily conveyed through artwork. Animation of learning objectives such as convulsions would better communicate important clues of a condition. Another common suggestion was a group game format or pairing players to encourage discussion among the community, transfer knowledge between generations, and assist with smartphone and touchscreen interfaces. Suggestions were documented for continued future development of the MANTRA serious game.

FCHVs revealed the serious game aided them in recalling prior knowledge and training on maternal and newborn health issues. The FCHV participants also said that the intervention strengthened their confidence in approaching communities, and supported their dedication to existing health programs [54, 60, 61].

The MANTRA game was well received by participants and stakeholders while recognizing that the game is still in the development stages. Participants said they enjoyed the game experience, felt motivated to continue playing or play again, learned from the game, and would like to share the game within the community.

## Discussion

In the broader picture of mHealth and public health, the exploratory study from MANTRA is an example of designing and implementing a serious game intervention as a method to educate and empower groups and communities. Insights from technical implementation and field evaluations of the iterative process may help adapt the design process for application to future educational game interventions.

### Strengths and transferability

MANTRA is the first study of its kind, developing a serious game through co-creation with a hard-to-reach low-literacy audience that was successfully evaluated with user feedback and knowledge gain from the game. This unique achievement was enabled by several principles and team strengths.

This unique study demonstrates the opportunities of truly multidisciplinary research efforts driven not by academic pursuit alone, but by the local need in the target area and target audience in an underprivileged country. Varied expertise brings together geoscience, computer science, disaster risk reduction, gender analysis, and maternal and child health to work on a common problem for social good. The serious game resulting from the development method benefits from this range of expertise, experience, and involvement of end users through co-creation with experts and the target audience.

The presented game development method is generic and therefore transferable to other digital interventions for low-literacy audiences. Pictogram sets can be switched out for newly designed pictograms that are contextualized and localized to other study areas, countries, or topics. Some participant suggestions looked beyond the purpose of a serious game, but are interesting in identifying wants and needs, such as adding functionalities like communications or call functions to provide support, advice, and information about existing health programs. These suggestions also highlight the potential for the serious game to expand into other functions to further support risk and health-seeking behaviors among low-literacy audiences and complement existing health programs.

### Limitations and challenges

Our pilot study of the MANTRA intervention has limitations and faced several challenges. Due to the exploratory nature of the pilot study and short time frame, participant selection targeted a specific audience and limited the geographic area of the study, therefore our sample is unlikely to be representative of the diverse population of Nepal. A further limitation is the possibility of a negative impact on women due to misunderstanding of the messages in the game, which could result in users internalizing the wrong information from the intervention. While researchers attempted to correct misunderstandings, in an educational environment, misunderstandings leading to unintentional decisions and negative consequences are not fully in the control of researchers.

The research team varied throughout the conception and 9 month duration of the project, ranging from six researchers and expanding to over 10 team members. As the MANTRA research team was dispersed and distant from the study area, it was not feasible to include end users in more than 2 cycles of formal feedback due to time and resource constraints. In light of these constraints for researchers as well as end users, the principle of co-design guides our actions and designs choices, but we understand it is not practical to co-create and co-design every decision needed to create a game. In the short time frame and with the limited resources available, we were not able to enlist a game developer in Nepal to assist with on the ground localization, contextualization, and field testing. If this had been possible, iterations of the design process could be rapidly completed, and the local cultural knowledge of the developer(s) would make the game easier for participants to understand, especially the geohazard content which was very new to participants.

Another consideration was designing a game that would be accessible to an already vulnerable target audience of low-literacy women. Clear visual communication and intuitive game interactions were high priorities to reach this already vulnerable sector, which constrained communication of learning objectives, progress tracking, and immediate feedback.

Field testing facilities were open community spaces, which posed difficulties for implementing a single user study with rigorous test conditions since participants chatted while playing. This challenging testing environment is well known among in-situ and semi-structured qualitative studies [61, 62]. Although rigorous user testing was not practical, in-situ testing provided valuable insights of how the intervention would actually be used in a facilitated educational group or as a stand-alone game to play at home. Far from being a challenge or limitation, the tendency to play together could remove barriers, such as lack of familiarity with a smartphone, and stimulate discussion about healthcare and geohazards, which could help to spread the educational messages of the intervention.

Although many problems were not apparent in the short duration of field evaluation sessions in the MANTRA pilot study, evidence from other studies demonstrates issues of implementing digital interventions. Problems with technology include the expense of devices and risk of theft, privacy concerns, capability to alter or delete software and data, and access to electricity, mobile networks, and internet. Problems with social dynamics include restrictions upon mobile phone use that may be imposed upon women and mobile phones being taken away or modified by family members [61, 63, 64]. These issues may create difficulties for end users themselves, within their household, and for implementation of interventions.

### Scale up

Scaling up a pilot intervention for public distribution could take several paths. A larger study with a greater number of participants could better represent diverse populations by randomly selecting participants from various regions in the study area. Involving a Nepalese game developer could accelerate the process of adjusting the game to the local audience by undertaking continued testing and development of the game to make it as contextualized as possible, and reduce time consuming communication between researchers in different time zones. Another pathway for scaling up is providing FCHVs with tablets to take to community workshops where the educational game intervention would be a facilitated group activity. An additional possibility is designing the game to be used in the home without a facilitator.

Several distribution issues are anticipated. One obstacle is incomplete coverage of mobile networks to download the game and update it regularly. Rather than requiring a formal distribution point and internet connection, the file could be shared from smartphone to smartphone via Bluetooth or a wired connection, although this approach has logistical challenges. Mobile coverage is growing, and projections suggest that soon most of the country could be covered by a mobile network [22, 56]. Another obstacle is interoperability between available hardware devices for large scale distribution.

Importantly, formal involvement of government stakeholders would integrate digital interventions into existing government public health programs. A consultative MANTRA workshop with government and non-governmental organization (NGO) stakeholders provided an opportunity to present the research team’s preliminary findings and gather feedback. Stakeholders recognized the value of the MANTRA serious game to engage and educate the public on vital health issues, and interest in further research was widespread. However, the stakeholders recognized that the game is ‘still a work in progress’ which would need more development to be understandable to all Nepalese users before being ready for public distribution.

### Game play in practice

Researchers were able to reach a target audience in a controlled environment in the community with smartphones brought by the study facilitators. This study in a controlled environment leaves some questions about reaching a target audience in everyday life [62]. Firstly, engagement remains a challenge. Even though participants enjoyed playing the games in facilitated sessions among their peers, would they continue playing at home for educational reasons when busy with their households? [61, 62]. Secondly, home use of the game intervention might increase women’s agency if they are able to cite the game as a source of reliable information and use this to negotiate healthcare access in traditional Nepali patriarchal households, where husbands and mothers-in-law often make decisions regarding healthcare [65, 66].

### Future work

Looking forward, future work would be field evaluation testing to represent a larger population by incorporating individuals across generations and genders while randomly selecting participants from various regions in Nepal. This expanded evaluation would build evidence and best practice for outreach and delivery of vital health information through educational games as public health interventions. Such future research could involve designing new modules such as nutrition, and also explore the potential for scale up, reaching and impacting target audiences, and effectively involving project partners and stakeholders in the co-design process. Producing behavior change is the ultimate purpose of the serious game intervention and remains a challenging open research question to be addressed by a longitudinal follow up study.

## Conclusions

Public health interventions for vulnerable populations in hard to reach settings in LMICs are rarely undertaken due to the cost, logistical difficulties and lack of scalability. Recently, mobile technology has begun to fill this gap by providing an infrastructure for reaching out to remote rural areas. Low-literacy and illiterate audiences are a challenging target group for digital mediums as they require brand new approaches when traditional text-based designs may not be appropriate.

In this paper, we described the MANTRA serious game development process for a public health intervention aimed at low-literacy women in remote rural communities in Nepal to improve their knowledge and healthcare seeking behavior around maternal, neonatal health and geohazards. This novel approach for a serious game as an mHealth intervention relies on several fundamental principles: a co-design process to develop culturally appropriate gamified mobile technology that is contextualized to the local population, pictograms-based artwork, user interfaces suitable for an illiterate audience, and evaluation with the target population.

Although the MANTRA serious game was designed for women in rural Nepal, insights from the developmental process could be transferable to other interventions and cultural or geographic settings. The development process itself is successful in that the resulting serious game for public health was engaging and enjoyable for the low-literacy target audience and participants identified with the setting of the serious game. These achievements in reaching a hard to reach low-literacy target audience in an LMIC setting support the MANTRA development process as a novel research endeavor with the potential for wider adoption and impact in public health interventions intended for low-literacy audiences in LMICs.

## Data Availability

The datasets used and/or analysed during the current study are available from the corresponding author on reasonable request for 10 years after publication.

## Abbreviations

LMICs: Low and middle income countries
FCHVs: female community health volunteers
HERD: Health Research and Social Development Forum
